# Chrysophanol Prevents Lipopolysaccharide-Induced Hepatic Stellate Cell Activation by Upregulating Apoptosis, Oxidative Stress, and the Unfolded Protein Response

**DOI:** 10.1155/2020/8426051

**Published:** 2020-07-04

**Authors:** Jiunn-Sheng Wu, Valeria Chiu, Chou-Chin Lan, Ming-Chieh Wang, I.-Shiang Tzeng, Chan-Yen Kuo, Po-Chun Hsieh

**Affiliations:** ^1^Division of Infectious Diseases, Taipei Tzu Chi Hospital, Buddhist Tzu Chi Medical Foundation, New Taipei City, Taiwan; ^2^Division of Physical Medicine and Rehabilitation, Taipei Tzu Chi Hospital, Buddhist Tzu Chi Medical Foundation, New Taipei City, Taiwan; ^3^Division of Pulmonary Medicine, Taipei Tzu Chi Hospital, Buddhist Tzu Chi Medical Foundation, New Taipei City, Taiwan; ^4^Department of Pharmacy, Taipei Tzu Chi Hospital, Buddhist Tzu Chi Medical Foundation, New Taipei City, Taiwan; ^5^Department of Research, Taipei Tzu Chi Hospital, Buddhist Tzu Chi Medical Foundation, New Taipei City, Taiwan; ^6^Department of Chinese Medicine, Taipei Tzu Chi Hospital, Buddhist Tzu Chi Medical Foundation, New Taipei City, Taiwan

## Abstract

Hepatic stellate cell (HSC) activation is a vital driver of liver fibrosis. Recent research efforts have emphasized the clearance of activated HSCs by apoptosis, senescence, or reversion to the quiescent state. LPS induces human HSC activation directly and contributes to liver disease progression. Chrysophanol is an anthraquinone with hepatoprotective and anti-inflammatory effects. This study aimed to investigate the pharmacological effects and mechanisms of chrysophanol in an LPS-induced activated rat HSC cell line (HSC-T6). The fibrosis phenotype was identified from the expression of *α*-smooth muscle actin (*α*-SMA), connective tissue growth factor (CTGF), and integrin *β*1 by western blot analysis. We examined DNA fragmentation by terminal deoxynucleotidyl transferase dUTP nick end labeling (TUNEL) staining. We detected the apoptotic markers p53 and cleaved caspase-3 by western blot analysis. Intracellular ROS were labeled with 2′,7′-dichlorofluorescein diacetate (DCF-DA) and the levels were measured by flow cytometry. Finally, we evaluated the ER stress markers binding immunoglobulin protein (BiP) and C/EBP homologous protein (CHOP) by Western blot analysis. Our results showed that chrysophanol decreased HSC-T6 cell viability in LPS-induced activated HSCs. Chrysophanol increased the expression of *α*-SMA, CTGF, integrin *β*I, p53, cleaved caspase-3, and DNA fragmentation. Chrysophanol also elevated ROS levels and increased the expression of BiP and CHOP. Pretreatment with chrysophanol prevented LPS-induced HSC-T6 cell activation by upregulating apoptosis, ROS accumulation, unfolded protein response (UPR) activation, and the UPR proapoptotic effect.

## 1. Introduction

Liver fibrosis is a global health burden that significantly elevates the risk of developing liver cirrhosis and hepatocellular carcinoma (HCC) [1]. Hepatic stellate cell (HSC) activation is considered to be a vital driver of liver fibrosis [2]. In normal liver, HSCs exist in a quiescent nonproliferative state, where they have a star-like shape with intracellular lipid storage droplets containing vitamin A as retinyl palmitate [2]. Activated proliferative myofibroblast-like phenotype HSCs result from liver injury and show specific changes, including upregulated proliferation, contractility, fibrogenesis, altered matrix degradation, chemotaxis, and inflammatory signaling [2]. These processes accumulate extracellular matrix (ECM) (including *α*-smooth muscle actin (*α*-SMA) and type I and III collagens), leading to liver fibrosis [2–4]. Multiple mechanisms are involved in the regulation of HSC activation: (1) fibrogenic and proliferative cytokines (transforming growth factor-beta 1 (TGF*β*1), platelet-derived growth factor (PDGF), vascular endothelial growth factor (VEGF), and connective tissue growth factor (CTGF)); (2) HSC-ECM interaction (integrin *β*1 and discoidin domain receptors (DDRs)); (3) the unfolded protein response (UPR); (4) oxidative stress; and (5) apoptosis signaling [2]. Several pharmacological agents have been demonstrated to target HSC activation, but none has been applied in clinical practice [2, 5]. Thus, other potential natural products with antifibrotic effects against HSC activation should be investigated.

Bacterial lipopolysaccharide (LPS) is the major component of Gram-negative bacteria. In patients with cirrhosis, relatively high levels of LPS are measured in portal, hepatic, and peripheral venous blood [6]. In both ligation of the common bile duct-(BDL-) induced cholestatic liver injury and carbon tetrachloride (CCl4) injection-induced toxic liver injury mouse models, translocation of bacteria and LPS across the intestinal epithelial barrier with increased permeability contributes to experimental liver disease progression [7]. LPS directly induces human HSC activation through the TLR4 signal transduction cascade to activate NF-*κ*B and JNK and then accumulates proinflammatory chemokines and adhesion molecules [8].

Dahuang, the root of *Rheum palmatum L*., is a well-known traditional Chinese herbal medicine that has been used to treat chronic liver disease and inflammation [9, 10]. Chrysophanol is an anthraquinone derivative isolated from the rhizomes of *R. palmatum L*. Previous studies demonstrated that chrysophanol shows anticancer, hepatoprotective, neuroprotective, anti-inflammatory, antiulcer, and antimicrobial activities [11–13]. Chrysophanol regulates the expression of various genes and proteins, such as GRP78, p-eIF2a, CHOP, caspase-12, Drp1, PTP1B, PAI-1, Bcl-2/Bax, and caspase-3 [13]. Chrysophanol also affects the NF-*κ*B, MAPK, PI3K/AKT, and PPAR-*γ* signaling pathways [11–13]. A previous study indicated that chrysophanol attenuates TGF-*β*1-induced HSC-T6 chemotactic migration [14]. Chrysophanol also shows protective effects against LPS/d-GalN-induced hepatic injury in mice through inhibition of the RIP140/NF-*κ*B pathway [15]. However, the pharmacological effects of chrysophanol and the underlying mechanisms in activated HSCs have not yet been reported.

Therefore, this study aimed to investigate the pharmacological effects and mechanisms of chrysophanol in LPS-induced activated rat HSC cells (HSC-T6), including fibrogenic factors, apoptosis, oxidative stress, and the UPR.

## 2. Materials and Methods

### 2.1. Reagents

Chrysophanol (CAS 481-74-3) and LPS (#L8774) were supplied by Sigma (MO, USA). The cell proliferation reagents WST-1 and RNase A were obtained from Roche Applied Sciences (Mannheim, Germany). 4′,6-Diamidino-2-phenylindole dihydrochloride (DAPI) was purchased from Thermo Fisher Scientific (MA, USA).

### 2.2. Antibodies

The antibodies used for immunofluorescence staining and Western blotting were as follows: rabbit polyclonal antibodies to *α*-SMA (ABclonal, MA, USA), p-JNK (ABclonal, MA, USA), JNK (ABclonal, MA, USA), p-p38 (Cell Signaling, MA, USA), p38 (Cell Signaling, MA, USA), p53 (Cell Signaling, MA, USA), cleaved caspase-3 (Cell Signaling, MA, USA), and GAPDH (ABclonal, MA, USA).

### 2.3. Cell Culture

HSC-T6, a rat HSC cell line, was purchased from Millipore (#SCC069, MA, USA). HSC-T6 cells were cultured at 37°C in Dulbecco's minimum essential medium (DMEM; Gibco, NY, USA) supplemented with 10% fetal bovine serum (FBS) and antibiotics (100 U/mL penicillin, 100 *μ*g/mL streptomycin, and 2.5 *μ*g/mL amphotericin B) in a humidified atmosphere with 5% CO_2_. The culture medium was replaced every other day. Once the cells reached 70%–80% confluency, they were trypsinized and seeded onto 6- or 24-well plastic dishes for further experiments. We used three replicates of 3–10 passages HSCs in each experiment.

### 2.4. Cell Viability Assay

Cell viability was measured using the WST-1 assay. Cells were seeded at a density of 5 × 10^4^ cells/mL in 24-well plates and cultured in phenol red-free DMEM containing 0.5% heat-inactivated FBS for 24 h. Cells were then incubated with the indicated concentrations (30 *μ*M) of chrysophanol for 24 h. The WST-1 reagent was added to the medium and incubated at 37°C for 2 h. The absorbance was measured at 450 nm with a microplate reader (Thermo Labsystems, MA, USA).

### 2.5. Measurement of Intracellular ROS Generation

Intracellular ROS generation was measured using the 2′,7′-dichlorofluorescein diacetate (DCF-DA; Sigma, MO, USA) reagent. The relative ROS level was measured by flow cytometry (BD Biosciences, CA, USA).

### 2.6. Western Blot

Cells were pelleted and resuspended in an ice-cold lysis buffer (20 mM Tris-HCl (pH 7.4), 150 mM NaCl, 1 mM EGTA, 1 mM NaF, 2 mM Na_3_VO_4_, 1 mM phenylmethylsulfonyl fluoride, 1% dilution of protease inhibitor cocktail (Sigma), and 1% Triton X-100). Samples were centrifuged at 14,000 g for 20 min at 4°C to yield cell lysates. Proteins were separated by gradient sodium dodecyl sulfate-polyacrylamide gel electrophoresis (SDS-PAGE) and transferred electrophoretically onto a nitrocellulose membrane. Immunoblotting was performed using specific primary antibodies and horseradish peroxidase-conjugated secondary antibodies (Cell Signaling, MA, USA), and peroxidase activity was evaluated using an enhanced chemiluminescence kit (Perkin-Elmer Life Science, MA, USA). The intensities of the reactive bands were analyzed using UVP Biospectrum (UVP, CA, USA).

### 2.7. Cell Morphology

The HSC-T6 cells were plated onto 6-well plates at an initial density of 1 × 10^5^ cells/ml to form a monolayer. Upon reaching 70%–80% confluency, the cells were treated under the following conditions: control, LPS (LPS-treated), Cho (chrysophanol-treated), and Cho + LPS (chrysophanol- and LPS-treated). Finally, cell morphology was observed from microscopic images taken under different conditions at several intervals and at three marked locations on each dish using a Nikon E400 phase-contrast microscope (Nikon, Tokyo, Japan).

### 2.8. Immunofluorescence Staining

HSC-T6 cells were fixed in 4% formaldehyde, blocked with PBS containing 2% FBS, and incubated with anti-*α*-SMA antibody (A7248, ABclonal). Subsequently, the cells were incubated with an FITC-labeled secondary antibody (Jackson Immunoresearch Laboratories). Finally, the cells were examined under an OLYMPUS IX 81 microscope (Olympus, Tokyo, Japan).

### 2.9. TUNEL Staining and Quantification

 Apoptosis-associated DNA fragmentation was visualized using a terminal deoxyribonucleotidyl transferase-mediated dUTP-digoxigenin nick end labeling (TUNEL) apoptosis detection kit (Roche, Mannheim, Germany). Cells were finally counterstained with DAPI and analyzed under an OLYMPUS IX 81 microscope (Olympus, Tokyo, Japan). Digital photographs were taken under fluorescence microscopy at 200× magnification. Twenty-five random high-power fields from each sample were chosen and blindly quantified [16].

### 2.10. Statistical Analysis

The statistical analysis was performed with IBM SPSS Statistics 25 (IBM, NY, USA). Data are expressed as the means ± standard deviation (SD). Groups were compared with one-way or two-way analysis of variance (ANOVA), followed by Bonferroni post hoc analysis. A value of *p* < 0.05 was considered to indicate statistical significance.

## 3. Results

To evaluate the effect of chrysophanol and LPS on HSC-T6 activation, we analyzed HSC-T6 treated with three different conditions: (1) control group (with neither chrysophanol nor LPS); (2) LPS group (treated with 3 *μ*g/mL LPS for 24 h); and (3) Cho + LPS group (pretreated with 30 *μ*M chrysophanol for 1 h and then treated with 3 *μ*g/mL LPS for 24 h).

### 3.1. Chrysophanol Prevented LPS-Induced Activation of HSC-T6 Cells

**Figure 1:**
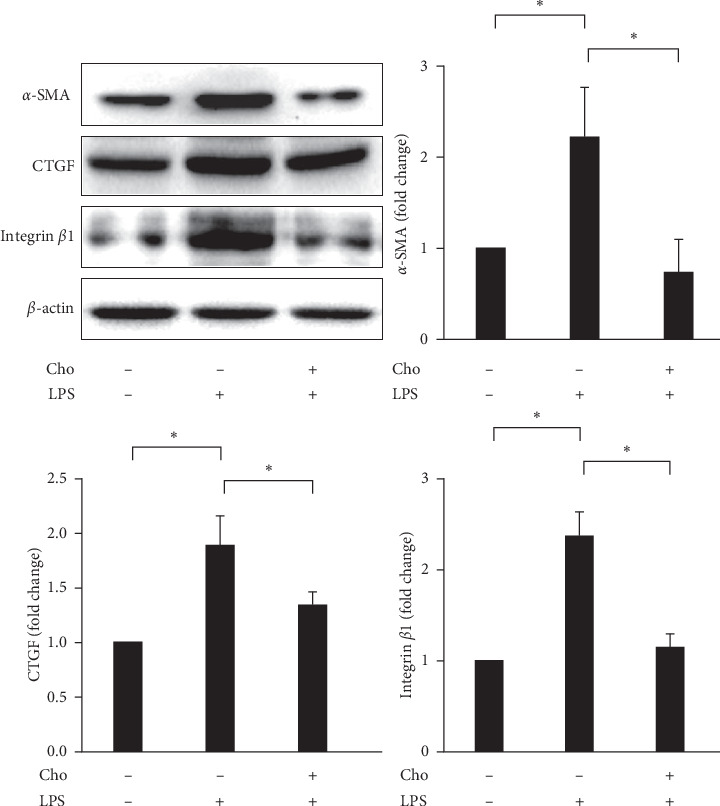
Chrysophanol (Cho) attenuated LPS-induced activated HSC-T6 cells. Changes in the expression of *α*-SMA, CTGF, and integrin *β*-1. *β*-Actin was used as an internal control. Quantitative results show the level of specific proteins assessed by ImageJ. All data are presented as mean ± SD. *n* = 3. ^*∗*^*p* < 0.05.

Elevated amounts of *α*-SMA characterize HSC activation [2]. We evaluated the expression of *α*-SMA by Western blot analysis. Our results indicated that LPS induced significantly increased expression of *α*-SMA in the LPS group compared with that in the control group (*p* < 0.05). The Cho + LPS group showed significantly decreased expression of *α*-SMA compared with the LPS group (*p* < 0.05) (Figure 1). The results suggested that chrysophanol pretreatment had a preventive effect on LPS-induced activation in HSC-T6 cells.

**Figure 2:**
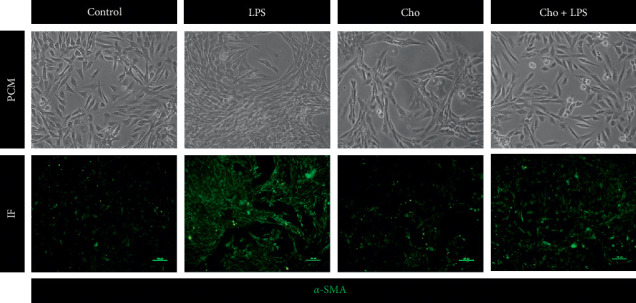
Effect of chrysophanol (Cho) and LPS on HSC-T6 cell morphology and *α*-SMA distribution. We observed cell morphology and *α*-SMA distribution using phase-contrast microscopy (PCM) and immunofluorescence (IF) staining, respectively, under the following conditions: control, LPS (LPS-treated), Cho (chrysophanol -treated), and Cho + LPS (chrysophanol and LPS-treated). Bar = 100 *μ*m. *n* = 3.

Phenotypically, quiescent HSCs have a relatively small cell body with cellular processes extending around the cell in a star-like configuration and characterized by a lack of *α*-SMA expression. Upon activation, HSCs are transformed into the activated phenotype, with a larger, flat, and clustering appearance and increased *α*-SMA expression [17, 18]. Our results show that the LPS group transdifferentiated into the activated phenotype observed by PCM and IF staining (*α*-SMA) compared with the control group. The Cho + LPS group showed a more quiescent-like phenotype (Figure 2).

### 3.2. Chrysophanol Decreased the Expression of CTGF and Integrin *β*-I in LPS-Induced Activated HSC-T6 Cells

During HSC activation and liver fibrogenesis, CTGF upregulated the expression, ECM production, migration, and proliferation of activated HSCs [19]. We evaluated the expression of CTGF by Western blot analysis. Our results showed that LPS induced significantly increased expression of CTGF in the LPS group compared with that in the control group (*p* < 0.05). The Cho + LPS group showed significantly decreased expression of CTGF compared with the LPS group (*p* < 0.05) (Figure 1).

Not only does the accumulation of ECM form a fibrotic construction but ECM components also interact with the collagen transmembrane receptor integrin. Integrins regulate the release and activation of TGF*β* and HSC activation [20]. Integrin receptors are composed of *α* and *β* subunits. Martin et al. demonstrated that integrin *β*1 regulates the profibrogenic phenotype of activated HSCs for the production of fibrotic ECM components, proliferation, contraction, and migration [21]. We evaluated the expression of integrin *β*1 by Western blot analysis. Our results indicated that LPS significantly increased the expression of integrin *β*1 in the LPS group compared with that in the control group (*p* < 0.05). The Cho + LPS group showed significantly decreased expression of integrin *β*1 compared with the LPS group (*p* < 0.05) (Figure 1).

### 3.3. Chrysophanol Decreased the Viability of HSC-T6 Cells Activated by LPS-Induction via Apoptosis

**Figure 3:**
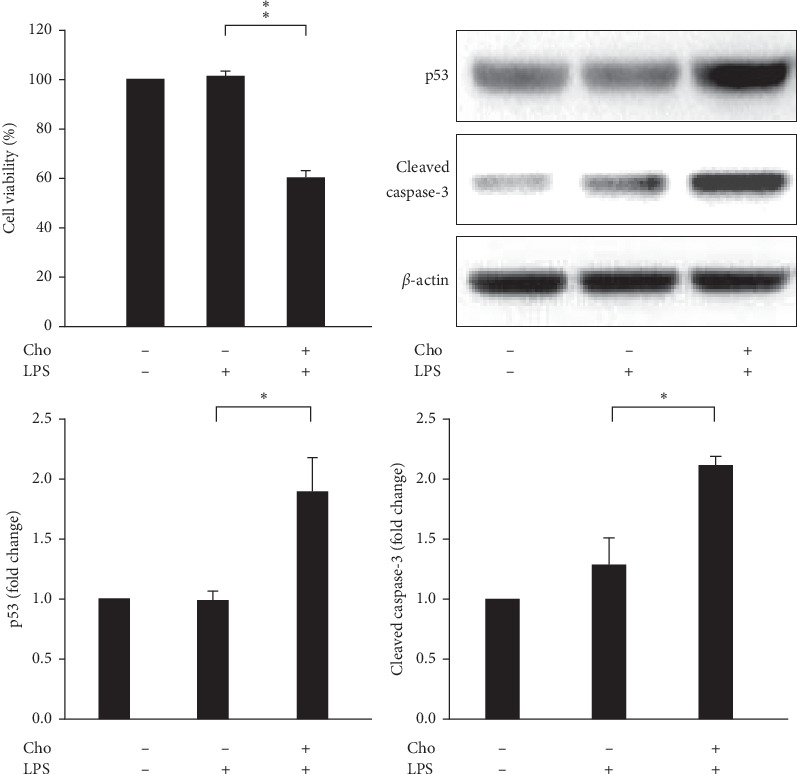
Chrysophanol (Cho) triggered cell death in HSC-T6 cells activated by LPS induction. Cell viability was determined using the WST-1 assay for three indicated groups. Changes in the expression of p53 and cleaved caspase-3 (active form of caspase-3). *β*-Actin was used as an internal control. Quantitative results showing the level of specific proteins assessed by ImageJ. All data are presented as mean ± SD. *n* = 3. ^*∗*^*p* < 0.05. ^*∗∗*^*p* < 0.01.

**Figure 4:**
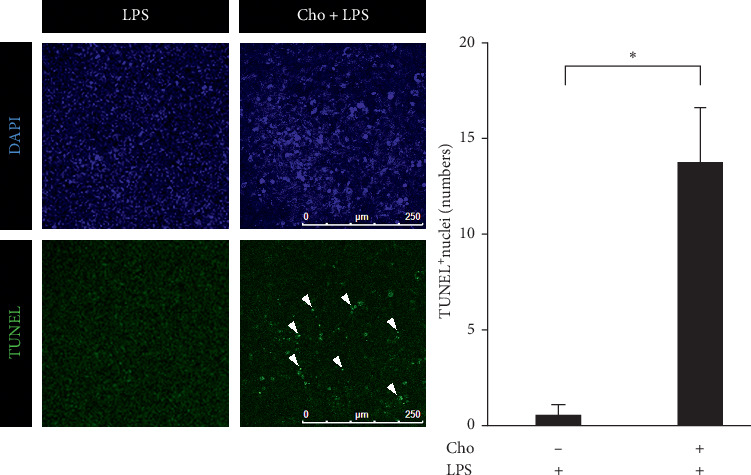
Chrysophanol (Cho) induced cell apoptosis in HSC-T6 cells activated by LPS induction assessed by TUNEL staining. Changes in nuclear morphology were visualized using TUNEL staining. The nuclei were counterstained with DAPI. Arrows indicate apoptotic phenomena by TUNEL staining. Quantitative results showing the TUNEL-positive cells. All data are presented as mean ± SD. *n* = 3. ^*∗∗*^*p* < 0.01.

Inducing apoptosis of HSCs during the resolution of liver fibrosis contributes to a reduction in the number of activated HSCs [5]. We evaluated the cell viability of HSC-T6 cells by using the WST-1 assay. The result showed significantly decreased cell viability in the Cho + LPS group compared with that in the LPS group (*p* < 0.01) (Figure 3). The expression levels of p53 and cleaved caspase-3 increased significantly in the Cho + LPS group compared with those in the LPS group (*p* < 0.05) (Figure 3). The results of TUNEL staining and the quantitation analysis showed significantly increased DNA fragmentation in the Cho + LPS group compared with the LPS group (*p* < 0.01) (Figure 4). These results suggested that chrysophanol decreased the cell viability of LPS-induced activated HSC-T6 cells via apoptosis.

### 3.4. Chrysophanol Elevated ROS Levels in HSC-T6 Cells Activated by LPS Induction

**Figure 5:**
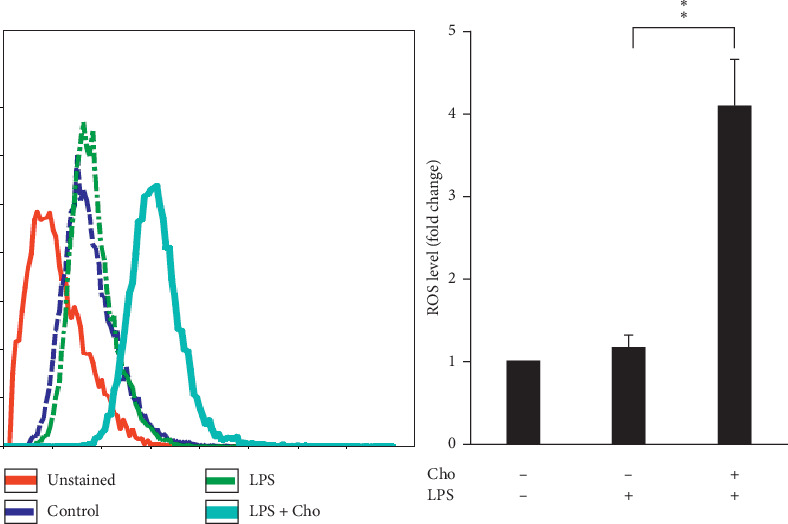
Chrysophanol (Cho) elevated ROS accumulation in HSC-T6 cells activated by LPS induction. The intracellular ROS level was determined by the DCF-DA assay, and the fluorescence was detected by FACS Calibur analysis. ROS generation is expressed as mean fluorescence intensity. All data are presented as mean ± SD. *n* = 3. ^*∗∗*^*p* < 0.01.

ROS has paradoxical effects on quiescent and activated HSCs. ROS produced by injured hepatocytes induces quiescent HSCs to transdifferentiate into the activated phenotype [2]. However, previous studies suggested that ROS accumulation triggers proapoptotic mechanisms in activated HSCs [22]. We detected ROS levels in HSC-T6 cells by using the DCF-DA assay. The results showed significantly increased ROS levels in the Cho + LPS group relative to the LPS group (*p* < 0.01) (Figure 5). We suggested that chrysophanol elevated ROS levels in LPS-induced activated HSC-T6 cells.

### 3.5. Chrysophanol Increased the UPR in LPS-Induced Activated HSC-T6 Cells

**Figure 6:**
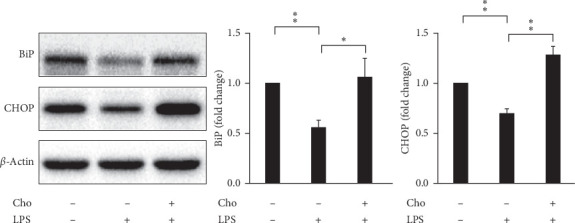
Chrysophanol (Cho) induced ER stress in LPS-induced activated HSC-T6 cells. Changes in the expression of BiP and CHOP. *β*-Actin was used as an internal control. Quantitative results showing the level of specific proteins assessed by ImageJ. All data are presented as mean ± SD. *n* = 3. ^*∗*^*p* < 0.05. ^*∗∗*^*p* < 0.01.

Increased expression of binding immunoglobulin protein (BiP) is a marker of UPR activation. When unfolded proteins accumulate, BiP disassociates from ER transmembrane transducers and induces UPR signaling cascades to alleviate ER stress. Excessive or prolonged ER stress can lead to apoptosis, which is promoted by the proapoptotic transcription factor C/EBP homologous protein (CHOP) [23]. We evaluated the expression of BiP and CHOP by Western blot analysis. Our results showed that the expression of BiP and CHOP significantly decreased in the LPS group compared with that in the control group (*p* < 0.01) (Figure 6). This change reflected the adaptation effect of activated HSCs to LPS. Both BiP (*p* < 0.05) and CHOP (*p* < 0.01) significantly increased in the Cho + LPS group relative to the LPS group (Figure 6). We suggest that chrysophanol increases UPR activation and proapoptotic effects in LPS-induced activated HSC-T6 cells.

### 3.6. Effects of Chrysophanol on HSC-T6 Cells

**Figure 7:**
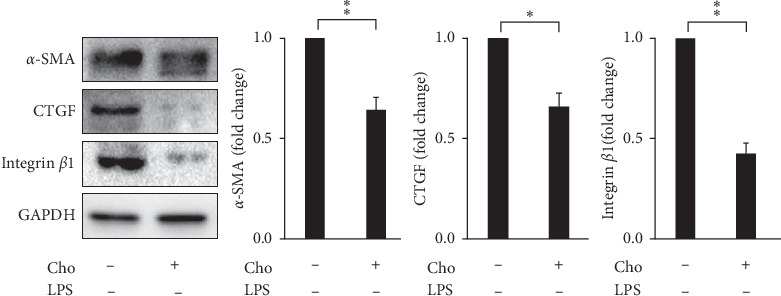
Chrysophanol (Cho) decreased the expression of *α*-SMA, CTGF, and integrin *β*-1 in HSC-T6 cells. Changes in the expression of *α*-SMA, CTGF, and integrin *β*1. GAPDH was used as an internal control. Quantitative results show the level of specific proteins assessed by ImageJ. All data are presented as the mean ± SD. *n* = 3. ^*∗*^*p* < 0.05.

**Figure 8:**
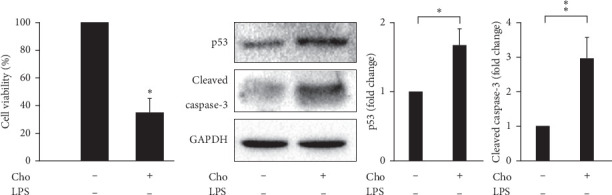
Chrysophanol (Cho) triggered cell death in HSC-T6 cells. Cell viability was determined using the WST-1 assay for three indicated groups. Changes in the expression of p53 and cleaved-caspase 3 (active form of caspase-3). GAPDH was used as an internal control. Quantitative results showing the level of specific proteins assessed by ImageJ. All data are presented as mean ± SD. *n* = 3. ^*∗*^*p* < 0.05. ^*∗∗*^*p* < 0.01.

**Figure 9:**
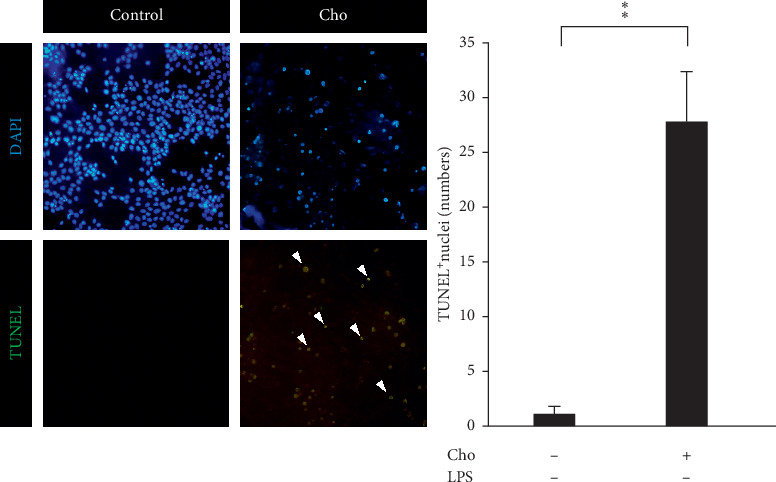
Chrysophanol (Cho) induced cell apoptosis in HSC-T6 cells assessed by TUNEL staining. Changes in nuclear morphology were visualized using TUNEL staining. The nuclei were counterstained with DAPI. Arrows indicate apoptotic phenomena by TUNEL staining. Quantitative results showing the TUNEL-positive cells. All data are presented as mean ± SD. *n* = 3. ^*∗∗*^*p* < 0.01.

**Figure 10:**
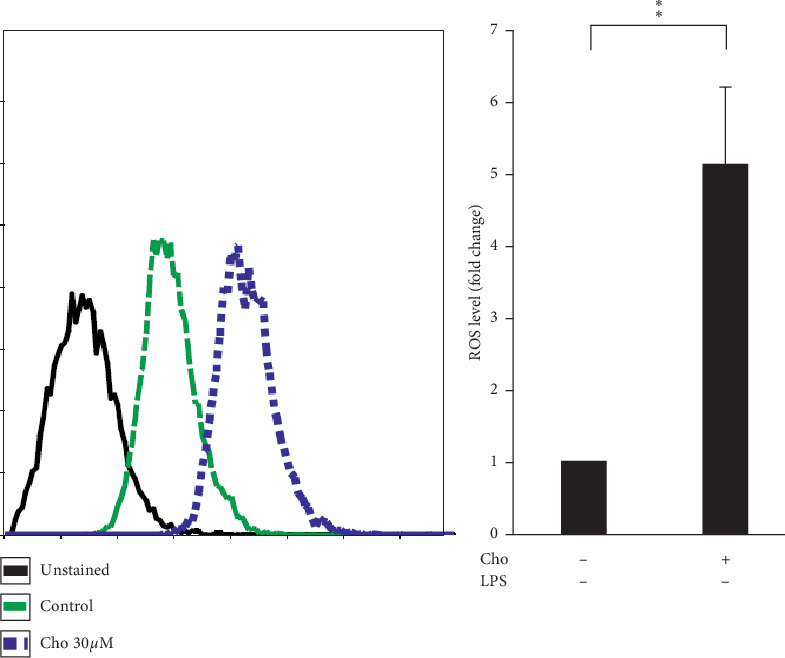
Chrysophanol (Cho) elevated ROS accumulation in HSC-T6 cells. (a) Intracellular ROS level was determined by the DCF-DA assay, and the fluorescence was detected by FACS Calibur analysis. ROS generation is expressed as mean fluorescence intensity. All data are presented as mean ± SD. *n* = 3. ^*∗*^*p* < 0.05.

**Figure 11:**
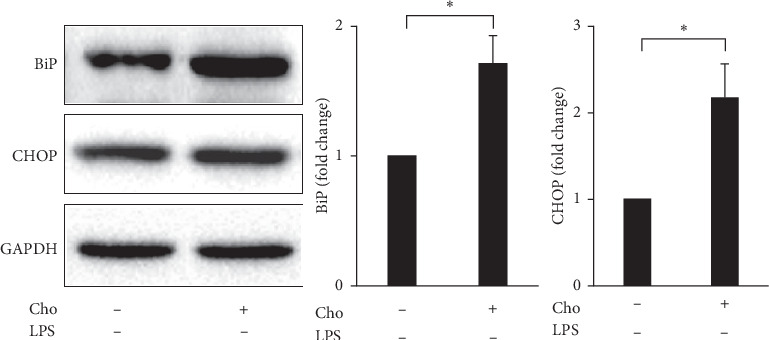
Chrysophanol (Cho) induced ER stress in HSC-T6 cells. Changes in the expression of BiP and CHOP. GAPDH was used as an internal control. Quantitative results showing the level of specific proteins assessed by ImageJ. All data are presented as the mean ± SD. *n* = 3. ^*∗*^*p* < 0.05.

Spatial and temporal heterogeneity of HSC activation increases with liver fibrosis progression [24]. Hence, we also investigated the effects of chrysophanol on HSC-T6 cells. Our results indicated that the effects of chrysophanol on HSC-T6 cells showed the same trend as on HSC-T6 cells activated by LPS induction. In HSC-T6 cells, chrysophanol significantly decreased the expression of *α*-SMA, CTGF, and integrin *β*-1 (Figure 7), decreased cell viability by apoptosis (Figures 8 and 9), elevated ROS accumulation (Figure 10), and increased UPR activation and the proapoptotic effect (Figure 11), compared with the control group. We suggest that chrysophanol has great potential to treat and prevent liver fibrosis based on the impacts of chrysophanol on both activated and nonactivated HSC-T6 cells.

## 4. Discussion

This study demonstrates that pretreatment with chrysophanol prevented LPS-induced HSC-T6 cell activation by upregulating apoptosis, ROS accumulation, and the UPR. To the best of our knowledge, this work is the first to investigate the inhibitory effects and underlying mechanisms of chrysophanol in HSCs activated by LPS induction.

Liver fibrosis is a dynamic and progressive process resulting from liver injury. HSC activation is known to be the primary mediator of liver fibrosis. Liver fibrosis is thought to be a reversible condition under proper treatment with pathogenic agents and control of HSC activation. Direct targeting of the required liver fibrosis agents or signal transduction pathways is a potential option to attenuate liver fibrosis [25]. However, regarding the complicated interactions involved in the microenvironments of activated HSCs, focusing on activated HSCs may inhibit liver fibrosis in a broader sense. Recently, increasing efforts have emphasized the clearance of activated HSCs once liver injury is alleviated. Clearance strategies for activated HSCs include, at the minimum, apoptosis, senescence, and reversion to the quiescent state [2]. Our results demonstrated that chrysophanol decreased the viability of LPS-induced activated HSC-T6 cells by apoptosis.

Mühlbauer et al. reported that LPS induces HSC activation through the Toll-like receptor 4 (TLR4), the NF-*κ*B signaling pathway, and the IL-8 activity [26]. LPS exposure increases proliferation, ROS generation, oxidative stress, and mRNA expression of *Collagen-1* and *α-SMA* of activated HSCs [27]. Compared with CCl_4_, CCl_4_ plus LPS injection promotes HSC activation through the elevated autophagy activity, retinoic acid signaling dysfunction, and indirect upregulation of TGF-*β* signaling, with increased *Col1a1*, *Acta2*, *Tgfb*, and *Timp1* mRNA expression and ACTA2/a-SMA and COL1A1 protein expression [28]. Our results demonstrated that LPS induced HSC activation with increased expression of *α*-SMA, CTGF, and integrin *β*-I, which were prevented by chrysophanol pretreatment.

Zhao et al. reported that chrysophanol inhibits the ER stress signaling pathways by downregulating UPR proteins (GRP78, p-elF2*α*, CHOP, and caspase-12) to attenuate cerebral ischemic/reperfusion injury [29]. Meanwhile, Park et al. demonstrated that chrysophanol activates proapoptotic proteins and increases intracellular Ca^2+^ levels, ROS generation, and ER stress by upregulating UPR proteins (PERK, eIF2*α*, GADD153, and IRE1*α*) to induce selective antiproliferative effects and apoptosis in breast cancer cells [30]. Regarding HSCs, ER stress overaccumulation in activated HSCs results in apoptosis mediated by calcium perturbations, the UPR, and expression of CHOP [31]. ER stress-induced activated HSC apoptosis is considered a potential therapeutic strategy for liver fibrosis [31]. Our results revealed that chrysophanol increased UPR activation (BiP) and proapoptotic effects (via CHOP) in LPS-induced activated HSC-T6 cells. Although chrysophanol seemed to show inverse regulation of the UPR under different conditions, the consequences suggest relatively healthy cell status and beneficial outcomes.

## 5. Conclusions

In summary, the present study demonstrated that pretreatment with chrysophanol prevented LPS-induced HSC-T6 cell activation (*α*-SMA, CTGF, and integrin *β*I) by upregulating apoptosis (p53, cleaved caspase-3, and DNA fragmentation), ROS accumulation, UPR activation (via BiP), and the UPR proapoptotic effect (via CHOP). These novel findings should deepen our understanding of the mechanical action of chrysophanol. Given that chrysophanol showed potential antifibrotic effects in activated HSCs, further in vivo studies are required to determine the possible effects on liver fibrosis models.

## Data Availability

The original data used to support the findings of this study are included within the article.
